# A Parameterized Leblond–Devaux Equation for Predicting Phase Evolution during Welding E36 and E36Nb Marine Steels

**DOI:** 10.3390/ma16083150

**Published:** 2023-04-17

**Authors:** Jun Fu, G. M. A. M. El-Fallah, Qing Tao, Hongbiao Dong

**Affiliations:** 1 School of Engineering, University of Leicester, Leicester LE1 7RH, UK; jf320@leicester.ac.uk; 2 Nanjing Iron & Steel United Co., Ltd., Nanjing 210044, China; 3 School of Materials and Physics, China University of Mining and Technology, Xuzhou 221116, China; qt15@le.ac.uk

**Keywords:** Leblond–Devaux equation, high heat input welding, marine steel, numerical simulation, heat-affected zone, phase evolution

## Abstract

High heat input welding can improve welding efficiency, but the impact toughness of the heat-affected zone (HAZ) deteriorates significantly. Thermal evolution in HAZ during welding is the key factor affecting welded joints’ microstructures and mechanical properties. In this study, the Leblond–Devaux equation for predicting phase evolution during the welding of marine steels was parameterized. In experiments, E36 and E36Nb samples were cooled down at different rates from 0.5 to 75 °C/s; the obtained thermal and phase evolution data were used to construct continuous cooling transformation diagrams, which were used to derive the temperature-dependent parameters in the Leblond–Devaux equation. The equation was then used to predict phase evolution during the welding of E36 and E36Nb; the quantitative experimental phase fractions of the coarse grain zone were compared with simulated results to verify the prediction results, which are in good agreement. When heat input is 100 kJ/cm, phases in the HAZ of E36Nb are primarily granular bainite, whereas for E36, the phases are mainly bainite with acicular ferrite. When heat input increases to 250 kJ/cm, ferrite and pearlite form in both steels. The predictions agree with experimental observations.

## 1. Introduction

In the shipbuilding and marine industries, the size of the steel structure has increased rapidly in recent years [1], and the time for welding thick steel plates can account for approximately 50% of the total construction time. To improve welding efficiency, high heat input welding methods have been widely used to weld thick steel plates for marine applications [2,3]. However, the heat-affected zone (HAZ) deteriorates under high heat input welding conditions [4]. This is due to the slow cooling rate and long residence time at elevated temperatures during high-heat-input welding [5]. Higher temperatures over a longer time in the HAZ lead to grain coarsening [6,7]. In addition, the coarse grain region is prone to forming coarse ferrite side plates, widmanstatten, upper bainite, and other abnormal microstructures during cooling [8]. As a result, the weld joint’s strength and hardness degrade, making it susceptible to brittle fracture, which is the most common failure mode and remains a major challenge for high-heat-input welding of thick steel plates for marine applications.

Experiments and modeling work have been carried out to overcome the challenge by optimising the welding parameters to avoid grains’ coarsening and brittle phases in HAZ. Bhadeshia et al. [9,10] examined the microstructure, grain size, and residual stress of HAZ to correlate the welding thermal cycle and phase transformation with the mechanical performance of the welded joints. These coupled fields are solved using FEA models, which are based on Leblond–Devaux equations [11] for phase evolution during welding. However, this model has not been applied to the high heat input welding of two marine steel plates, E36Nb with niobium and E36 without niobium.

Li et al. [12] and Ni et al. [13] implement Zener–Hillert type kinetics equations to predict the phase transformation TTT and CCT diagrams for steel, and the predicted CCT diagram was found to be in good agreement with those reported in the literature. However, these studies did not determine the predicted phase fraction of HAZ. Åkerström [14] developed a model to predict the austenite decomposition based on Kirkaldy’s rate equations [15] for boron steel, taking into account the effect of the chemical composition of the steel. However, the model showed a poor prediction at a high cooling rate. Aarne [16] developed a computational model based on the Johnson–Mehl–Avrami–Kolmogorov equation to simulate the austenite to bainite and martensite transformations. To increase accuracy, Hu et al. [17] predicted the microstructure of Q345 steel HAZ based on 3 SH-CCT diagrams under different peak temperatures; however, Hu acknowledged that the prediction of microstructure and hardness for actual welding HAZ is complex, and accuracy should be improved.

For E36 marine steel, Nb reduces the impact toughness of HAZ during the thermal welding cycle [18], and the effect of niobium on the mechanical properties of HAZ with high heat input is unclear. However, to obtain good quality and lower production costs, adding Nb as a microalloying element is widely used to improve the mechanical properties of marine steel [19]. A previous study has shown that as Nb concentration increases, the austenite grains become finer [20]. Typically, the addition of 0.01 wt.% Nb can enhance the strength to around 20 MPa for low-alloy carbon steel made by the thermo-mechanical control process (TMCP) [21]. According to the DNV-GL rules for classification, the minimum Nb concentration for a marine application is 0.01 wt.% [22]. Therefore, the effect of Nb on both the strength and impact toughness of HAZ should be considered in high-heat input welding. The impact toughness of HAZ is defined by microstructural evolution during welding. It is now essential to understand the continuous cooling transformation behaviour of HAZ and develop a corresponding simulated HAZ continuous cooling transformation (SHCCT) diagram for marine steels to investigate the effect of Nb on the phase evolution of HAZ. It will provide a valuable technical reference for high-heat input welding of marine steel.

**Figure 1:**
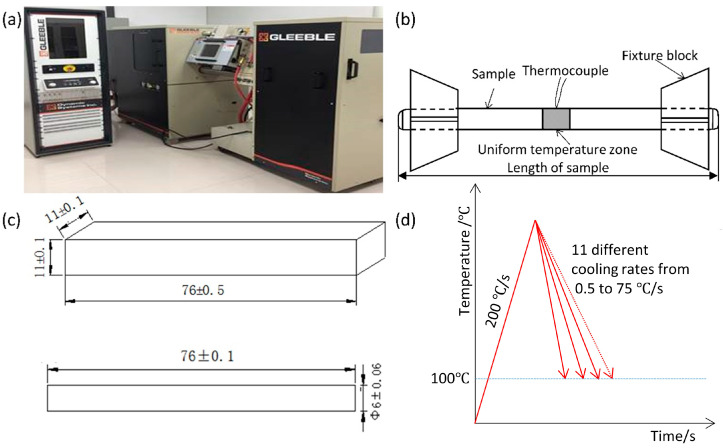
(**a**) Thermal-Mechanical Physical Simulation System; (**b**) schematic diagram of the sample during an experiment; (**c**) specimens’ geometry for the HAZ coarse grain zone experiment and SH-CCT experiment; and (**d**) heating and cooling profiles of SH-CCT experiments.

In this study, we carried out a series of purpose-designed experiments in a Thermal-Mechanical Physical Simulation System (Figure 1a) under controlled heating and cooling conditions akin to high heat input welding to simulate (1) HAZ continuous cooling transformation (SH-CCT) and (2) grain coarsening. Two widely used steel grades, E36Nb with niobium and E36 without niobium, were simulated with heat inputs of 100 kJ/cm and 250 kJ/cm, respectively. Detailed results on ferrite, pearlite, bainite, and martensite phases were reported during high-heat input welding of the two types of steel. The Leblond–Devaux (L–D) equation and Koistinen–Marburger (K–M) equation parameters for the phase evolution model were determined. The thermal-phase evolution FEA models are verified by comparing them against experimental observations of the coarse grain zone. Moreover, a parameterized model based on L–D and K–M equations was provided for predicting ferrite, pearlite, bainite, and martensite phase fractions during the welding thermal cycle process.

## 2. Materials and Methods

Two thick marine steel plates were rolled from a continuous casting slab. The chemical compositions of the two plates are listed in Table 1.

A thermocouple was spot-welded in the middle of the sample to measure temperature (Figure 1b). The specimen’s size for SH-CCT development is Φ 6 mm × 76 mm, and the specimen’s size for the coarse-grain zone experiment is 11 mm × 11 mm × 76 mm (Figure 1c). The hardness of simulated samples for SH-CCT development was measured after polishing and etching using a Struers Vickers hardness tester at a load of 300 g.

Heat treatments were carried out as schematically illustrated in Figure 1d to simulate the HAZ continuous cooling transformation (SH-CCT) experiment. Samples were heated to 1320 °C, which is the estimated peak temperature during high heat input welding of thick E36 plates. Then the austenitic structure was cooled to 100 °C at 11 different cooling rates: 0.5 °C/s, 1 °C/s, 1.5 °C/s, 2 °C/s, 5 °C/s, 10 °C/s, 15 °C/s, 20 °C/s, 30 °C/s, 50 °C/s, and 75 °C/s, and then freely cooled down to room temperature. During the cooling, austenite decomposes to form ferrite, pearlite, bainite, and/or martensite. The resulting phase composition depends on the temperature history, chemical composition, and austenite grain size.

The parameters for simulating grain coarsening in HAZ are listed in Table 2. The cycles involve heating at 180~200 °C/s to 1350 °C/s, holding for 1~2 s (the holding time at peak temperature becomes longer with the increase of heat input [5]), and then cooling at prescribed rates through the temperature range from 800 to 500 °C, corresponding to △t_8/5_ = 71.2 s and 444.8 s; the cooling rates are 4.23 °C/s and 0.67 °C/s. Different Δt_8/5_ times correspond to heat outputs of 100 and 250 kJ/cm, respectively.

The transversely cut sample, which was thermally simulated and connected to the thermocouple at the centre, was used as the microstructure observation surface. To prepare a metallographic sample at room temperature, mechanical polishing was performed, followed by etching with a 4% nitric acid alcohol solution. The microstructure of the etched samples was examined using a metallographic microscope.

## 3. Leblond–Devaux and Koistinen–Marburger Equations for Phase Transformations during Welding

The thermal cycle during the welding process induced austenite formation and decomposition of HAZ. The study defined the temperature-dependent metallurgical phase transformations involved in this process and computed the heterogeneous phase composition in the HAZ. During continuous heating, austenite forms at Ac_1_ ≈ 720 °C, and the sample becomes fully austenitic at Ac_3_ ≈ 880 °C. The peak temperature in the HAZ is heated to around 1320 °C and then cooled to room temperature during the welding process. As the temperature decreases, the austenite decomposes into ferrite, pearlite, bainite, and martensite.

The material properties of the steel plate are temperature- and phase-dependent, consisting of a mixture of phases, each with a phase fraction that is evolving during the model. The material’s thermal conductivity and mass density were calculated using phase fraction weighting.

JMatPro is a material properties simulation software developed by Sente that can be used to calculate various properties of metal materials [23,24]. The physical properties calculation module of JMatPro was used to calculate density, thermal conductivity, Poisson’s ratio, and Young’s modulus as temperature-dependent functions of the steel plate according to its chemical composition.

The ferrite, pearlite, and bainite transformation rates are computed by the Leblond–Devaux equation [11]. The model primarily considers carbon-diffusion-based phase transformations that occur in steel during its thermal history. The phase evolution of the thermal simulation was composed of two analysis procedures: nonlinear heat transfer instantaneous and nonlinear austenite decomposition processes [3].

Leblond and Devaux proposed evolution equations for phase transformations during welding using the following form:

(1) $$\xi^{d}=K_{s\to d}\left(T\right)\xi^{s}-L_{s\to d}{(T)\xi }^{d},$$

where $\xi^{s}$ is the proportion of the source phase, $\xi^{d}$ is the proportion of the destination phase, $K_{s\to d}\left(T\right)$ and $L_{s\to d}(T)$ represents the temperature-dependent functions. The L–D equation assumes that the rate of reaction is proportional to the deviation from equilibrium and, therefore, asymptotically achieves the equilibrium fraction as time increases. The model has been widely used to predict phase evolution, where the phase transformation on cooling occurs at a different temperature. The formulation calls for temperature-dependent functions that specify the phase transformation properties. This study selected the general coefficients of the Leblond–Devaux equation to model the transformations, so the parameters $K_{s\to d}\left(T\right)$ and $L_{s\to d}(T)$ should be determined by experimental data in the form of a CCT diagram to derive the fitting constants. Initially, Kirkaldy and Venugopalan [15] proposed models that predict CCT diagrams for steels. Then, the model by Kirkaldy made reasonable predictions for HAZ microstructures in welds [13,25].

The Koistinen–Marburger [26] model is used to model the transformation of austenite into martensite:

(2) $$\xi^{d}=-\xi^{s}\beta T,$$

The amount of undercooling below the so-called martensite start temperature $M_{s}$ controls the volume fraction of martensite. The martensite-start temperatures for E36 and E36Nb were calculated using Jamtpro to be 433.1 °C and 432.1 °C, respectively. 

A value for the Koistinen–Marburger coefficient *β* is empirical. However, it can be correlated to the temperature at which the martensitic transformation is considered complete if the integrated form of the phase transformation model is used [27,28,29]. The SH-CCT curve is validated with the experimental SH-CCT curve, and the study obtained the *β* to be 0.00004 (1/k) for the E36 marine steel.

Assuming a constant cooling rate, the phase fraction of retained austenite is $\xi_{0}^{s}$, at the volume fraction of martensite $\xi^{d}$. Below $M_{s}$ the amount of formed martensite is proportional to the undercooling below $M_{s}$, given by $M_{s}-T$, the rate equation, which can be integrated to:

(3) $$\xi^{d}=\xi_{0}^{s}(1-\exp\left(-\beta \left(M_{s}-T\right)\right)),$$

## 4. Results and Discussion

### 4.1. Phase Evolution of E36 and E36Nb in Experiments at Different Cooling Rates

**Figure 2:**
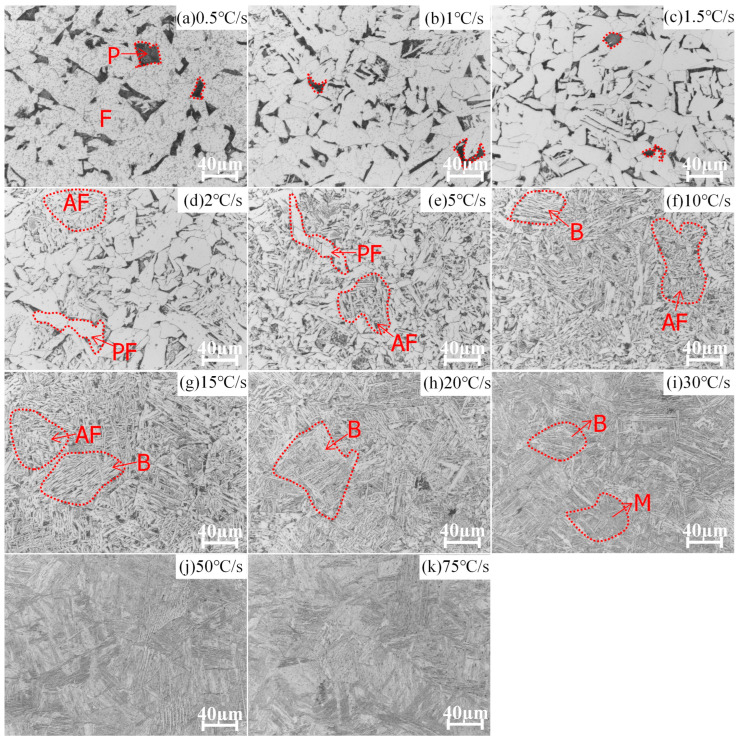
Optical microscope images of E36 microstructures with 11 different cooling rates as indi-cated during the thermal cycle of SH-CCT experiments. B stands for bainite, M for martensite, P for pearlite, F for ferrite, AF for acicular ferrite, and PF for proeutectoid ferrite.

Figure 2 shows HAZ microstructures in E36 cooled at 11 different cooling rates. The present study employs the ImageJ software for the quantification of phase fractions based on the grayscale intensity of each respective phase [30,31,32]. The ImageJ software employs a methodology that measures the fraction of a specific phase area in relation to the total area present in optical microscope images. The approach is comprised of several steps: Firstly, colours are employed to differentiate phases that possess similar colouration. Secondly, a threshold is set to extract the specific colour area, and the corresponding pixel value of the designated area is obtained. Finally, the entire area is selected, and the pixel value of the entire area is measured. The fraction of pixels present in the designated area is then divided by the total number of pixels in the entire area to calculate the phase fraction of the specific region.

Phases and their fractions are summarised in Table 3. When the cooling rate changes from 0.5 to 1.5 °C/s, phases are ferrite (F) (84–90 %) and pearlite (P) (16–10 %); acicular ferrite appears when the cooling rate changes from 2 °C/s to 20 °C/s; as the cooling rate increases from 30 °C/s to 75 °C/s, martensite (M) forms and there is no acicular ferrite.

**Figure 3:**
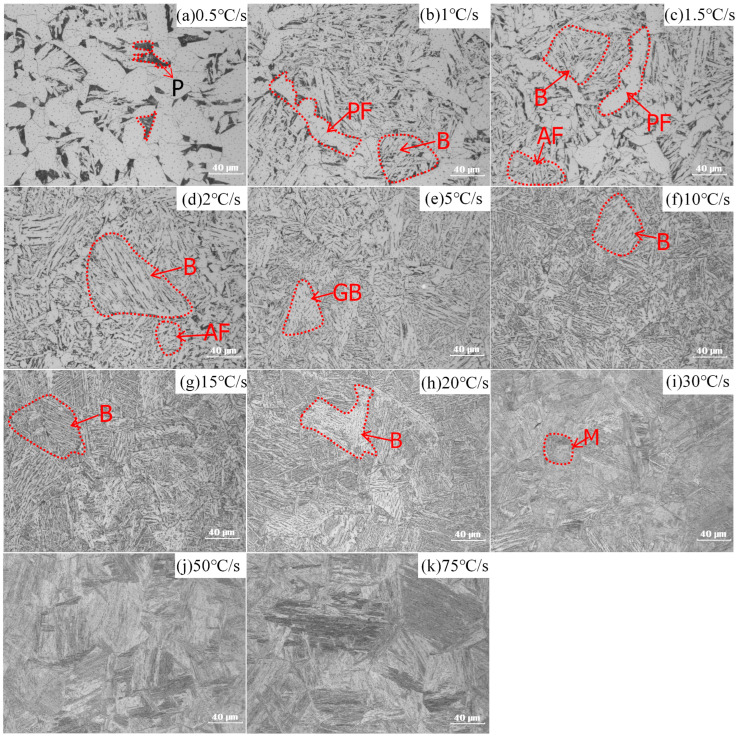
Optical microscope images of E36Nb microstructures with 11 different cooling rates as indicated during the thermal cycle of SH-CCT experiments. B stands for bainite, GB stands for granular bainite, M for martensite, P for pearlite, F for ferrite, AF for acicular ferrite, and PF for proeutectoid ferrite.

Figure 3 shows phase evolution of E36Nb cooled at 11 different cooling rates; the phases are ferrite (F) (86%) and pearlite (P) (14%) at a cooling rate of 0.5 °C/s. The acicular ferrite appears when the cooling rate changes from 1 °C/s to 2 °C/s; as cooling rates increase above 15 °C/s, bainite (B) and martensite (M) structures form. Phases and their fractions are summarised in Table 3 for different cooling rates of E36Nb.

Table 3 summarises phases formed at different cooling rates of E36 and E36Nb. The phase fractions are calculated by ImageJ software according to the grayscale of each phase. The effect of Nb on the phase evolution of HAZ is noticeable at high heat input; the addition of Nb inhibits ferrite transformation and promotes bainite formation, which is associated with the solid solution of Nb [33,34]. Another key point is the acicular ferrite, which determines the impact toughness during high-heat input welding [35]. Yang et al. [6] discovered that effective micron-level inclusion produces the acicular ferrite nucleation tendency. However, Nb tends to segregate towards the interfaces between the micron-level inclusions and matrix, which has a negative effect on the nucleation potential of acicular ferrite. Therefore, the acicular ferrite forms at broader cooling rates for E36 steel and a narrower cooling rate for E36Nb steel. Niobium reduces the range of cooling rates for acicular ferrite transformation. The acicular ferrite is formed when the cooling rate changes from 1 °C/s to 2 °C/s for E36Nb and from 2 °C/s to 20 °C/s for E36.

### 4.2. SH-CCT Diagrams and Temperature-Dependent Parameters of Leblond–Devaux Equation for E36Nb

**Figure 4:**
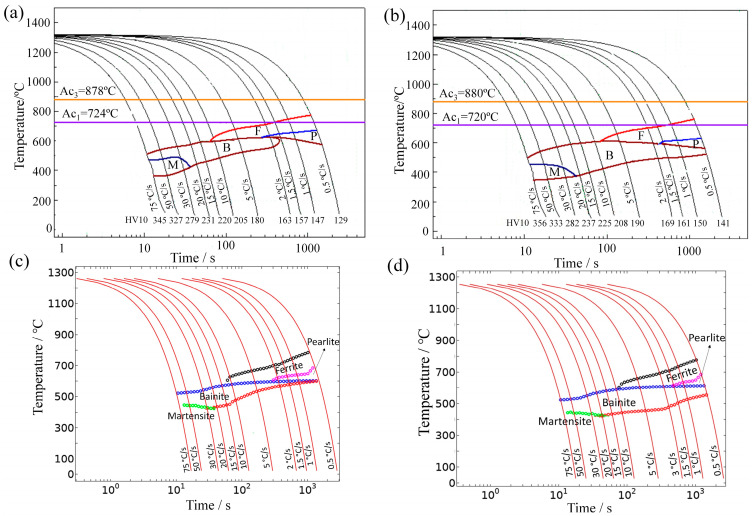
Continuous cooling transformation curves of E36 and E36Nb, where F, P, M, and B represent the phase-formed zones for ferrite, pearlite, martensite, and bainite at a given cooling rate. (**a**,**b**) Experimental SH-CCT diagrams of E36 and E36Nb using a Thermal-Mechanical Physical Simulation System with the corresponding hardness measurements. (**c**,**d**) Simulated SH-CCT diagrams of E36 and E36Nb, respectively, using COMSOL software.

Figure 4a,b shows SH-CCT diagrams of E36 and E36Nb based on the phase evolution under a series of cooling rates. The figures also indicate the hardness measurements (HV10) of both steels, cooled at 11 different rates. The Ac_1_ and Ac_3_ temperatures measured were 724 °C and 878 °C for E36 and 720 °C and 880 °C for E36Nb. With increasing heat input, the hardness decreases for E36 and E36Nb; with Nb addition, the hardness increases in the range of 3–12 kg/mm^2^ due to the microstructure change. The microstructure is mainly martensite with bainite under very fast cooling rates from 30 °C/s to 75 °C/s; this changes to ferrite with pearlite under slow cooling rates from 0.5 °C/s to 10 °C/s. The effect of niobium addition on the phase evolution of E36 steel can be seen in Figure 4a,b. Niobium expands the bainite transformation area, slightly reducing the ferrite and pearlite transformation areas. The bainite formed at cooling rates from 1.5 °C/s to 75 °C/s for E36 and larger cooling rates from 0.5 °C/s to 75 °C/s for E36Nb, which was the dominant feature of continuous cooling transformation for E36Nb.

Figure 4c,d shows the simulated SH-CCT diagrams of E36 and E36Nb using COMSOL software. Determining Leblond–Devaux model parameters $K_{s\to d}\left(T\right)$ and $L_{s\to d}(T)$ are complicated for specific steel grades. $K_{s\to d}\left(T\right)$ and $L_{s\to d}(T)$ take the form of a temperature-dependent interpolation function as the input parameter of the model. The parameters determine the phase transformation during the welding thermal cycle process, where the thermal expansion experiments measure Ac_1_ and Ac_3_. The phase transformations are intrinsically coupled, and it is not easy to treat one phase transformation separately from another. Therefore, a simulated SH-CCT diagram is best compared to an experimental SH-CCT diagram that includes all relevant phase transformations. The FEA method was used to simulate SH-CCT diagrams and then compared to the experimental version to calibrate and verify the temperature-dependent functions that describe each phase transformation. The austenite decomposition module of the simulation is based on the Leblond–Devaux equation. By adjusting the $K_{s\to d}\left(T\right)$ and $L_{s\to d}(T)$ constantly, the Leblond–Devaux model parameters were determined, and the temperature-dependent functions describing this transformation are given in Table 4 and Table 5.

The $K_{s\to d}\left(T\right)$ and $L_{s\to d}(T)$ of austenite to ferrite, bainite, and pearlite are the key input boundary conditions of the phase evolution model. The SH-CCT diagram shows the time and temperature when a phase begins to form, given a cooling rate. The cooling curves are shown in Figure 4c,d, corresponding to cooling rates of 75 k/s to 0.5 k/s. In this case, a cooling rate of 5 k/s causes bainite to form at a temperature of 610 °C. Either bainite or martensite is generated at a rate of 75 k/s. Therefore, this extremely rapid cooling would be used in practise to generate a martensitic structure.

## 5. Application of Parameterized Leblond–Devaux Equation

### 5.1. Predicted Phase Evolution of E36 and E36Nb with High Heat Inputs Using Parameterized Leblond–Devaux Equation

**Figure 5:**
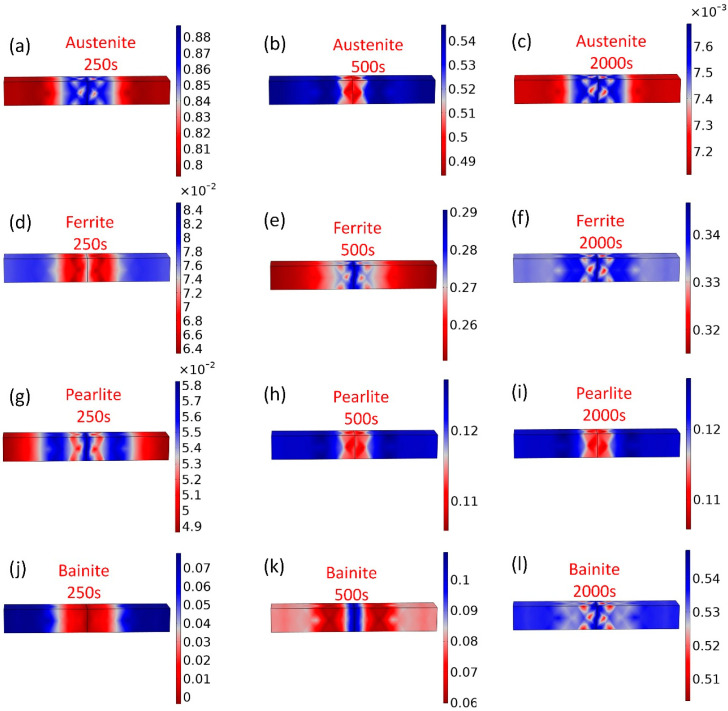
Predicted phase evolution for E36Nb with a heat input of 250 kJ/cm at different welding times as indicated. (**a**–**c**) Fraction of austenite; (**d**–**f**) fraction of ferrite; (**g**–**i**) fraction of pearlite; and (**j**–**l**) fraction of bainite.

The adjusted Leblond–Devaux parameters were implemented in the model to predict the microstructural evolution of the coarse grain zone during thermal experiments. Figure 5 shows the evolution of the phases for E36Nb with a heat input of 250 kJ/cm at different welding times of 250 s, 500 s, and 2000 s, including the fractions of austenite, ferrite, pearlite, and bainite. As a result, austenite decomposition and ferrite, pearlite, and bainite gradually formed at different times.

**Figure 6:**
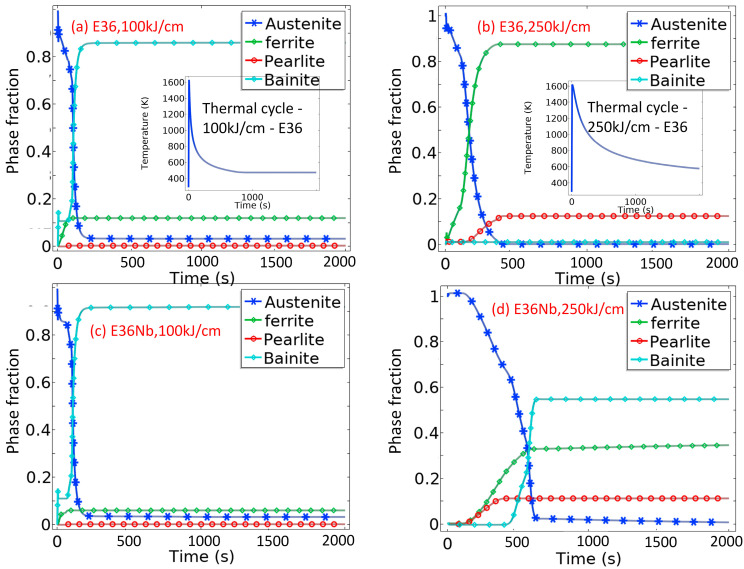
Predicted phase evolution of selected points during thermal welding cycle by COMSOL software; (**a**,**b**) E36 with 100 kJ/cm and 250 kJ/cm heat inputs; (**c**,**d**) E36Nb with 100 kJ/cm and 250 kJ/cm heat inputs.

In this study, the thermal cycle with different heat inputs and adjusted L–D and K–M parameters was implemented in the model to predict the phase transformation of the coarse grain zone with 100 kJ/cm and 250 kJ/cm heat input, as shown in Figure 6. The centre point of the sample has been chosen to calculate the thermal cycle curve of the coarse grain zone during the thermal experiment. The quantitative experimental phase fractions of the coarse grain zone are compared with the simulated results listed in Table 6 to verify the prediction results, which are in good agreement.

### 5.2. Phases in HAZ of E36 and E36Nb Welded with Heat Inputs of 100 kJ/cm and 250 kJ/cm

In thermal experiments to simulate grain coarsening, different heat input means different cooling rates during the cooling stage of the welding thermal cycle process, and cooling rates affect the microstructures of HAZ. Compared with faster cooling (heat input of 100 kJ/cm), the microstructure of HAZ with slower cooling (heat input of 250 kJ/cm) extends the time for prior austenite grain growth [36], resulting in a rabid coarsening of grain size in HAZ [37]. Peak temperatures (1350 °C) in the HAZ close to the fusion boundary are significantly higher than the Ac_3_ temperature of E36 steels, leading to the coarsening of austenite grains.

**Figure 7:**
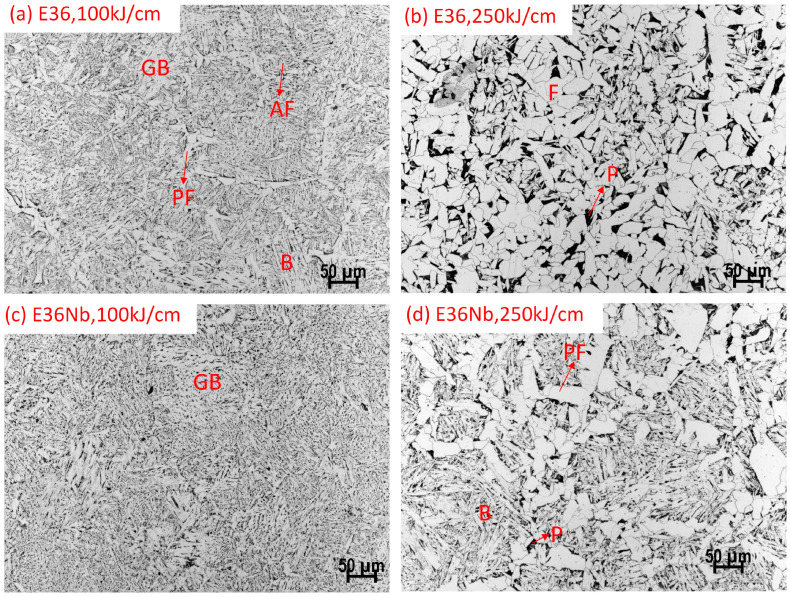
Phases in the coarse-grain zone in HAZ for E36 and E36Nb welded with different heat inputs of 100 and 250 kJ/cm. B stands for bainite, P for pearlite, F for ferrite, GB for granular bainite, AF for acicular ferrite, PF for proeutectoid ferrite.

Figure 7 shows the coarse-grain zone (HAZ) microstructure for E36 and E36Nb; the microstructure of HAZ is mainly bainite for E36 and granular bainite for E36Nb at 100 kJ/cm (cooling rate, 4.23 °C/s). With an increase in heat input, the microstructure of E36 becomes mainly ferrite, while the microstructure of E36Nb is proeutectoid ferrite and bainite when the heat input is increased to 250 kJ/cm (cooling rate is 0.67 °C/s).

The cooling rate curve of 4.23 °C/s mainly runs through the B-phase-formed zones of E36 and E36Nb, as shown in the SH-CCT diagrams (Figure 4), and the cooling rate curve of 0.67 °C/s mainly runs through the F-phase-formed zones of E36 and the F and B-phase-formed zones of E36Nb. The phase types of the experimental coarse-grain zone agree with the model predictions in Figure 4.

## 6. Conclusions

A new numerical model was used to predict the phase evolution of HAZ in E36 and E36Nb welding marine steels with a heat input of 100 kJ/cm and 250 kJ/cm by using a parameterized Leblond–Devaux equation and the Koistinen–Marburger equation. The model predicts the thermal cycle and phase fraction of marine steels. These parameters are achieved by adjusting the experimental SH-CCT diagram.

1. Niobium addition on E36 marine steel reduces the cooling rate range of acicular ferrite transformation. The acicular ferrite formed within broader cooling rates for E36 steel from 2 °C/s to 20 °C/s and a narrower cooling rate for E36Nb steel from 1 °C/s to 2 °C/s;
2. Phases in HAZ of E36Nb welded with a high heat input of 100 kJ/cm consist of acicular ferrite, proeutectoid ferrite, and bainite; phases in HAZ of E36 contain granular bainite and pearlite. Phases in HAZ of E36, welded with a high heat input of 250 kJ/cm, are ferrite and pearlite, but phases in HAZ of E36Nb are proeutectoid ferrite and bainite;
3. Leblond–Devaux equation parameters of $K_{s\to d}\left(T\right)$ and $L_{s\to d}(T)$ for both steels have been evaluated and simulated as temperature-dependent functions. A parameterized model based on the Leblond–Devaux and Koistinen–Marburger equations is provided for the thermal welding cycle to predict the phase fraction of marine steels.

## Figures and Tables

**Table 1:** Chemical composition of E36 and E36Nb steel plate, wt.%.

Steel	C %	Si %	Mn %	S %	P %	Nb %	Ti %	Al %
E36	0.08	0.21	1.51	0.002	0.012	/	0.016	0.03
E36Nb	0.08	0.25	1.52	0.002	0.013	0.012	0.016	0.03

**Table 2:** Coarse zone simulation parameters of E36 and E36Nb.

Peak Temperature °C	Heating Rate °C/s	Holding Time s	Δt_8/5_ s	Heat Input kJ/cm
1350	200	1.0	71.2	100
	180	2.0	444.8	250

**Table 3:** Phase fraction of HAZ with 11 different cooling rates. In the table, F stands for ferrite, P stands for pearlite, PF stands for proeutectoid ferrite, B stands for bainite, AF stands for acicular ferrite, and M stands for martensite.

Cooling Rate °C/s	Steel Grade	F %	P %	PF %	B %	AF %	M %
0.5	E36	84	16	-	-	-	-
	E36Nb	86	14	-	-	-	-
1	E36	86	14	-	-	-	-
	E36Nb	-	3	55.5	40	1.5	-
1.5	E36	90	10	-	-	-	-
	E36Nb	-	-	15	80	5	-
2	E36	-	6	60	30	4	-
	E36Nb	-	-	-	99	1	-
5	E36	-	10	40	35	15	-
	E36Nb	2	-	-	98	-	-
10	E36	-	-	15	35	50	-
	E36Nb	5	-	-	95	-	-
15	E36	-	-	-	60	40	-
	E36Nb	-	-	-	100	-	-
20	E36	-	-	-	90	10	
	E36Nb	-	-	-	96	-	4
30	E36	-	-	-	80	-	20
	E36Nb	-	-	-	70	-	30
50	E36	-	-	-	70	-	30
	E36Nb	-	-	-	60	-	40
75	E36	-	-	-	50	-	50
	E36Nb	-	-	-	20	-	80

**Table 4:** Temperature-dependent parameters of austenite decomposition for E36Nb.

Temperature/°C	Austenite to Ferrite		Austenite to Bainite		Austenite to Pearlite	
	*K* (1/s)	*L* (1/s)	*K* (1/s)	*K* (1/s)	*L* (1/s)	*L* (1/s)
0	0	0	0	0	0	0
300		0				0
400						
450						
470						
520	0		0.05		0	
540			0.005			
560			0.005			
580				0.002		
600	0		0.005		0	
620			0	0.0002		
640	0.0012				0.0004	
650		0.0002			0.00004	0.0002
710	0.00017					
800		0.002				0.0002
1000	0.002	0.002			0	0.002

**Table 5:** Temperature-dependent parameters of austenite decomposition for E36.

Temperature/°C	Austenite to Ferrite		Austenite to Bainite		Austenite to Pearlite	
	*K* (1/s)	*L* (1/s)	*K* (1/s)	*L* (1/s)	*K* (1/s)	*L* (1/s)
0	0	0	0	0	0	0
300		0				0
400						
440						
460						
470						
520	0		0.05		0	
540			0.005			
560			0.005			
580				0.002		
590			0.005			
600	0				0	
610			0			
620				0.0002		
640	0.002				0.0007	
650		0.0002			0.00004	0.0002
710	0.0025					
800		0.002				0.0002
1000	0.002	0.002			0	0.002

**Table 6:** The microstructure fraction of coarse grain zone on the experiment and simulation results.

Steel	Heat Input kJ/cm	Ferrite Fraction %		Pearlite Fraction %		Bainite Fraction %	
		Experiment	Simulation	Experiment	Simulation	Experiment	Simulation
E36	100	12.1	12.1	/	/	87.9	86.1
	250	85.9	87.7	14.1	12.5	/	1.1
E36Nb	100	4.2	6.1	/	/	95.8	91.9
	250	38.6	34.6	9.7	11.2	51.7	54.9

## Data Availability

For further datasets, please contact the corresponding author.
